# Hookworm infection associates with a vaginal Type 1/Type 2 immune signature and increased HPV load

**DOI:** 10.3389/fimmu.2022.1009968

**Published:** 2022-10-18

**Authors:** Millicent A. Omondi, Eya H. Kamassa, Gnatoulma Katawa, Christèle N. Tchopba, Celina Vogelbusch, Marijo Parcina, Edlom P. Tchadié, Oukoe M. Amessoudji, Kathrin Arndts, Simplice D. Karou, Yaovi Ameyapoh, Malewé Kolou, Achim Hoerauf, Laura E. Layland, William G. C. Horsnell, Manuel Ritter

**Affiliations:** ^1^ Wellcome Centre for Infectious Diseases Research in Africa (CIDRI-Africa), Institute of Infectious Disease and Molecular Medicine (IDM), Department of Pathology, Division of Immunology, Faculty of Health Science, University of Cape Town, Cape Town, South Africa; ^2^ Unité de Recherche en Immunologie et Immunomodulation (UR2IM)/Laboratoire de Microbiologie et de Contrôle de Qualité des Denrées Alimentaires (LAMICODA), Ecole Supérieure des Techniques Biologiques et Alimentaires, Universite de Lomé, Lomé, Togo; ^3^ Institute for Medical Microbiology, Immunology and Parasitology (IMMIP), University Hospital Bonn (UKB), Bonn, Germany; ^4^ German-West African Centre for Global Health and Pandemic Prevention (G-WAC), Partner Site Bonn, Bonn, Germany; ^5^ German Centre for Infection Research (DZIF), Neglected Tropical Disease, Partner site Bonn-Cologne, Bonn, Germany; ^6^ Institute of Microbiology and Infection, University of Birmingham, Birmingham, United Kingdom

**Keywords:** helminths, hookworm, sexually-transmitted diseases, HPV - human papillomavirus, Immune modulation, type 1 and type 2 immunity

## Abstract

Helminth infection-driven changes to immunity in the female reproductive tract (FRT) is an immune axis that is currently understudied but can have major implications for the control of FRT infections. Here we address how human hookworm infection associates with vaginal immune profile and risk of Human papillomavirus (HPV) infection. Stool, blood, cervical swabs and vaginal flushes were collected from women from the Central region of Togo to screen for hookworms (*Ancylostoma duodenale)* and high carcinogenic risk HPV types, *via* Kato Katz and PCR, respectively. Cytokine, chemokine and immunoglobulin levels were analysed in cervicovaginal lavages and plasma samples. A pronounced mixed Type 1/Type 2 immune response was detected in the vaginal fluids of women with hookworm infection and this immune signature was a notable feature in hookworm-HPV co-infected women. Moreover, hookworm infection is positively associated with increased risk and load of HPV infection. These findings highlight helminth infection as a significant risk factor for acquiring a sexually transmitted viral infection and potentially raising the risk of subsequent pathology.

## Introduction

Soil-transmitted helminth (STH) infections in low- and middle-income countries (LMIC) are common (1). Disproportionately high incidence and mortality (>85%) of Human Papillomavirus (HPV)-associated cervical cancer (CC) also occur in LMIC (2).

Helminth infections can result in diverse changes in the ability of the host to control viral infection. For example, CD8^+^ T cell responses against murine norovirus are reduced in mice infected with *Trichinella spiralis* (3). Similarly, reactivation of gamma herpesvirus, MHV68, occurs in *Heligmosomoides polygyrus*-infected mice due to raised IL-4 and STAT6 impairing IFN-γ dependent antiviral immunity (4). In contrast, expansion of virtual CD8^+^ T memory cells in *Schistosoma mansoni* exposed mice enhances control of murine gamma herpesvirus, MuVH4 (5). Helminth infections alter the risk of vaginal viral infection and pathology. STH infections have been reported to increase the risk of HPV infection in South America and in Togo (6, 7). Direct effects of helminth infection on the female reproductive tract (FRT) following *Schistosoma haematobium* eggs transiting the urogenital tract cause significant pathology (8) and increase the risk of HIV infection (9–11). Additionally, *Wuchereria bancrofti* can raise the risk of HIV, possibly due to increased FRT recruitment of CD4^+^ T cells (12).

Helminth infections that do not colonise or transit the urogenital tract or associated lymphoid organs significantly alter urogenital immunity against viral infection/pathology. For example, co-infections of *Nippostrongylus brasiliensis* – Herpes simplex virus type 2 (HSV-2), which is suggested as a co-factor in the HPV-driven etiology of invasive cervical cancer (13), results in increased viral-induced pathology that is driven by parasite-initiated vaginal IL-5, IL-33 and eosinophil responses in mice (14). A small number of human studies support this finding. Gravitt et al. (6) reported that STH infections were associated with a 60% increased HPV prevalence in women in South America and identified this to be associated with raised type 2 cytokines -in vaginal washes. In addition, our group recently deciphered distinct sociodemographic factors and helminth infections as risk factors associated with FRT infections and observed in detail that hookworm infections were associated with an increased risk of HPV infection (7). However, systemic and vaginal immune profiles were not analysed and the immune mechanisms underlying this observation remain uncertain and need further evaluation. Thus, in the present study, we investigated local vaginal immune parameters associated with hookworm-HPV co-infection and expand on existing work by investigating the association between hookworm infection and increased viral high carcinogenic risk HPV-DNA load.

## Methods

### Ethics statement

Participants were recruited from the Central region of Togo in 2019 as part of a German Research Foundation (DFG)-funded GSAT study within the German-African Cooperation Projects in Infectiology. All participants were over 18 years of age at the time of recruitment and gave written informed consent prior to sample collection. Ethical approval was received from the Comité Bioéthique pour la Recherche en Santé (CBRS) of the Ministry of Health of Togo (N°26/2017/CBRS) and the Ethics Committee at the University Hospital Bonn, Bonn, Germany (Lfd. Nr. 273/16).

### Study population

Samples were collected from study participants from six villages (Sakalaoudè, Tcheve, Fazao, Sagbadahi, Aleheridè and Kikimini) in the Central region of Togo in October 2019. To obtain a more comprehensive overview of the patient cohort, an epidemiological-based survey was conducted at the beginning of the study to assess sociodemographic variables, sexual behavior and vaginal hygiene practices (7). A total number of 367, sexually active and premenopausal women, aged between 18-56 years was recruited. Pregnant and/or HIV-positive women were excluded from the study. HIV positive women were directed to a local clinic for treatment.

### Parasitological examination

Examination for parasitic eggs (*Ancylostoma duodenale*, *Hymenolepis nana*, and *Schistosoma mansoni*) performed in stool samples using the Kato-Katz technique as previously described (15). Presence of *Schistosoma haematobium* eggs in urine samples was established by centrifuging urine (10 ml) at 1500 rpm for 5 minutes and examining the resulting pellet microscopically, but no *S. haematobium* eggs could be detected. To determine the presence of *Onchocerca volvulus* microfilariae, two skin snips of 1-2 mm in diameter were taken from left and right iliac crests using biopsy forceps. Skin snips were then incubated in 100 µl of NaCl (0.9%) in 96-well microtiter plates for 18-24 hours at room temperature. Then, the presence of microfilariae was examined microscopically at 40x magnification. However, no *O. volvulus* microfilariae were detected. Moreover, blood smears were either incubated in water for 15 minutes, fixed with methanol and stained with 10% Giemsa for 45 minutes or fixed with May-Grunwald solution for 2 min and then stained with a 1:10 diluted Giemsa staining solution for 15 min to microscopically assess blood-dwelling microfilariae and *Plasmodium* spp., respectively. Staining reagents were obtained from Química Clínica Aplicada (QCA, Tarragona, Spain) and microscopic assessment of parasites was performed using the Olympus CX23 light microscope (Olympus, Rungis, France). All parasitological analyses were directly performed in the field or in the laboratory of the UR2IM in Lomé, Togo.

### Diagnosis of HPV infection

To assess HPV infection, cervical swabs were transferred in eNAT medium (Copan, Brescia, Italy) and shipped to the IMMIP, Bonn, Germany. Then, DNA was extracted from cervical swabs using the Seegene Microlab Nimbus IVD automaton kit (Seegene Inc., Seoul, Republic of Korea) according to the manufacturer’s instructions to screen for HPV using the CFX96 IVD Real-time PCR System (Bio-Rad Laboratories Inc., Feldkirchen, Germany). The Anyplex™ 2 HPV HR Detection (CE/VD) kit was used to detect HPV 16, HPV 18, HPV 31, HPV 33, HPV 35, HPV 39, HPV 45, HPV 51, HPV 52, HPV 56, HPV 58, HPV 59, HPV 66 and HPV 68. The test was performed in a diagnostic reference laboratory for microbiology. This diagnostic assay was chosen because of its extensive clinical and analytical evaluation through VALGENT-3 Framework (16).

### Analysis of HPV intensity

To assess if hookworm infection influences HPV viral load, we applied a semi-quantitative analysis using three categories (“intensity grade 1-3”). This type of semi-quantification is part of IVD/CE-approved diagnostic based on the proprietary chemistry of the SeeGene. In detail, extracted DNA is added to the mastermix and instead of the endpoint thermal cycler programme, a cyclic programme was chosen. The mastermix chemistry for HPV typing is based on high-resolution-melt profiling, the semi-quantification is based on the high-resolution melt (HRM) detection and the intensity of the obtained peaks after 30^th^ (intensity grade 3), 40^th^ (intensity grade 2) and the end-cycle (50th cycle; intensity grade 1) is based on the proprietary diagnostic (CE/IVD) algorithm. In addition, this assay uses internal control to assure that just the samples of certain cellularity are diagnostically valid and the same target gene is used as the inhibition control.

### Collection of plasma and vaginal flush

Blood was collected in 5 ml EDTA tubes and centrifuged at 4000 rpm for 5 minutes. Then, plasma was collected and stored at -20°C. In addition, vaginal flushes were collected by flushing the vagina of the enrolled women with 1 ml sterile PBS. To remove the cells, VF were centrifuged at 1200 rpm for 5 minutes and supernatants were stored at -20°C. Then, plasma and VF samples were shipped to Bonn, Germany for analyses of soluble cytokines, chemokines and antibodies.

### Assessment of cytokine, chemokine and immunoglobulin levels in the vaginal flush

Levels of Type 1 cytokines (IFN-γ, TNF-α, IL-12p70 and IL-18), proinflammatory cytokines (IL-6 and IL-1β), Type 2 cytokines (IL-5, IL-4 and IL-13), cell survival and growth factors (IL-2 and GMCSF), as well as chemokines (MIP-1α, MIP-1β, MCP-1, SDF-1α, GRO-α, eotaxin, IP-10, RANTES and IL-8) in the plasma and vaginal flush samples were measured using the ProcartaPlex Human Th1/Th2 & Chemokine Panel 1 Luminex assay (Thermo Fisher Scientific, Vienna, Austria). In addition, IL-17A, IL-21, IL-22, eotaxin-2 and eotaxin-3 were also analysed using a Human ProcartaPlex Mix&Match kit (Thermo Fisher Scientific). Moreover, levels of IgA, IgE, IgM, IgG1, IgG2, IgG3 and IgG4 were measured using the human antibody isotyping 7-Plex ProcartaPlex Panel (Thermo Fisher Scientific). All Luminex assays were analysed on the Luminex™ MAGPIX system (Luminex, Tokyo, Japan). In addition, IL-10 was measured by high sensitivity enzyme-linked immunosorbent assay (ELISA) using the High Sensitivity IL-10 Human ELISA Kit (Thermo Fisher Scientific) according to the manufacturer’s instructions. To normalise cytokine, chemokine and immunoglobulins levels relative to the protein concentration in the VF, protein levels were measured using the Bio-Rad Protein Assay Dye reagent concentrate (Bio-Rad Laboratories, Hercules, USA). Individual analyte concentration was then expressed as ratio of the measured parameter and the total protein concentration of the sample.

### Statistical analyses

Statistical analyses were performed using the PRISM 8 program (GraphPad Software, Inc., La Jolla, USA). Before testing for statistical significances between the groups, we performed a D’Agostino-Person omnibus normality test to test the distribution of the values. Since variables were non-parametric, a Mann Whitney U test was used to compare two groups, whereas a Kruskal-Wallis-test was performed to compare more than two groups. If a Kruskal-Wallis test was significant, a Dunn’s multiple comparison test was performed for a further comparison of the groups. To assess whether *Plasmodium* infection was associated with hookworm or HPV infection status, a Chi-square test was applied. To test the relationship between HPV intensity and immune parameters, a Spearman’s correlation test was performed and in order to determine whether hookworm infection was a risk factor for the severity of HPV infection, we performed a univariate logistic regression analysis with HPV intensity as a dependent factor. All associations, correlations and regression analysis were performed using the SPSS software Version 27.0 (IBM, New York, USA) and odds ratio (OR) was calculated with a 95% confidence interval and a p-value < 0.05 was considered as significant.

## Results

### Study population characteristics

**Figure 1:**
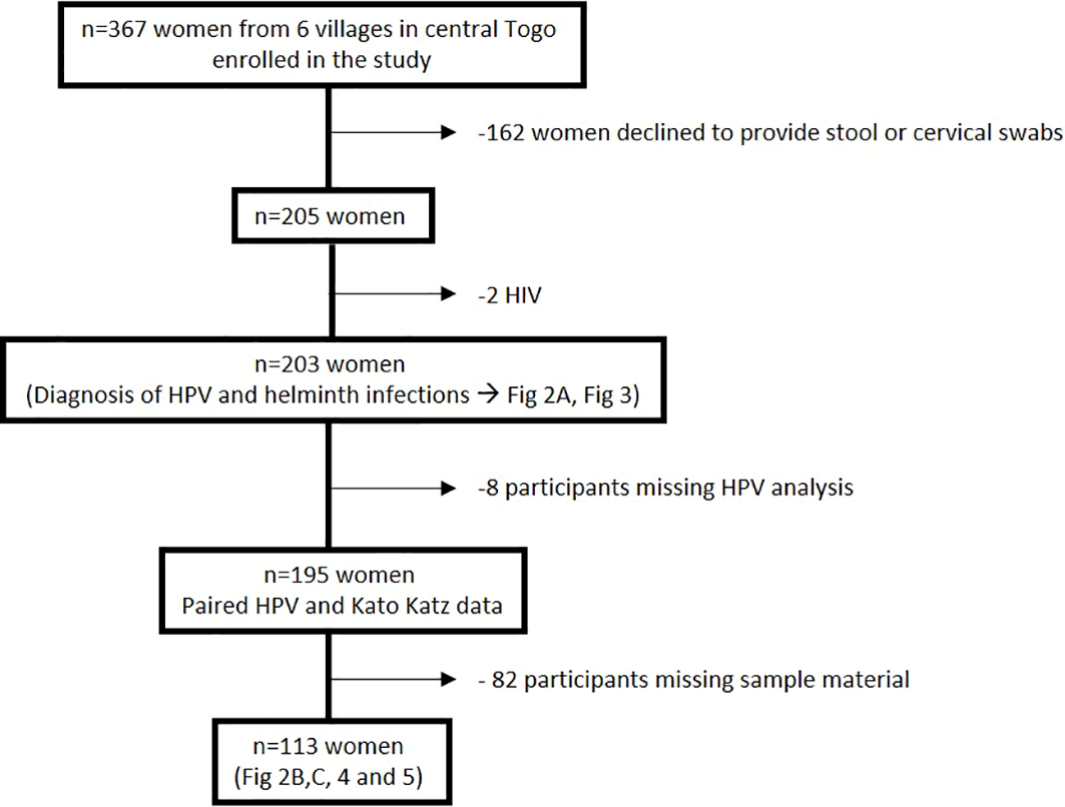
Overview of the study cohort from six villages in the Central region of Togo.

367 women were enrolled in this study of which 205 women consented to provide paired stool samples and cervical swabs. Two women tested positive for HIV and were excluded from the study, while 8 participants had missing HPV data. Hookworm prevalence was assessed in 203 women and HPV infection prevalence could be investigated in 195 women. However, sufficient amounts of plasma and vaginal flush (VF) samples could be obtained only from a study cohort of 113 women to perform the immunological analyses (**Figure 1**). Enrolled women were 18-56 years old with a median age of 32 years and 94.1% of participants were married, 99.5% were non-smokers, and 88.2% did not consume alcohol. Moreover, 57.1% of participants reported the use of hormonal contraceptives, 42.9% had no education, and 66% earned less than 27 dollars a month (**Table 1**). Data on sexual practices and vaginal hygiene practices associated with the risk of sexually transmitted diseases revealed that harsh vaginal cleaning, including washing with soap, detergent and/or antiseptic, were common, with 42.8% of participants reporting these practices (**Table 2**). Moreover, harsh intimate washing using finger, sponge or other material were reported by 58.6% of women, whereas only 8.4% of the women introduced products intravaginally. With regards to sexual practices, 69.5% of participants reported having had only one sexual partner in their lifetime, 14.8% reported consistent condom use, 52.2% reported sexual debut age between 10 and 20 years, 41.9% of the women reported that their partner has multiple partners, and 2.5% engaged in paid intercourse.

**Table 1:** Characteristics of enrolled women.

Characteristics	Number (n=203)	%
**Age [years]**		
18-25	54	26.6
26-32	49	24.1
33-39	52	25.6
>40	48	23.6
**Marital status**		
Married	191	94.1
Single	3	1.5
In a relationship	4	2
Divorced	2	1
Widowed	3	1.5
**Smoker**		
Yes	1	0.5
No	202	99.5
**Alcohol consumption**		
Yes	24	11.8
No	179	88.2
**Hormonal contraceptive use**		
Yes	116	57.1
No	84	41.4
N/A	3	1.5
**Level of education**		
Primary School	76	37.4
Secondary school	24	11.8
Other	14	6.9
No	87	42.9
Not reported	2	1
**Monthly income [US Dollar]**		
<27	134	66
27-62	10	4.9
62-124	2	1
No income	33	16.3
Do not know	24	11.8

**Table 2:** Sexual and vaginal hygiene practices of enrolled women.

Characteristics	Number (n=203)	%
**Number of partners**		
0	1	0.5
1	141	69.5
2	52	25.6
3	5	2.5
4	1	0.5
Not known	3	1.5
**Condom use**		
Every time	30	14.8
Most of the time	5	2.5
Never	137	67.5
Sometimes	28	13.8
Not reported	3	1.5
**Partner has multiple partners**		
Yes	85	41.9
No	104	51.2
Don't know	8	3.9
Not reported	6	3.0
**Paid intercourse**		
Yes	5	2.5
No	196	96.6
Not reported	2	1.0
**Age of first intercourse [years]**		
10-15	22	10.8
16-20	84	41.4
21-25	14	6.9
>25	1	0.5
Don't know	62	30.5
Not reported	20	9.9
**Vaginal cleaning practices**		
Water	50	24.6
Soap/detergent	9	4.4
Water and soap	69	34
Water, soap and antiseptics	9	4.4
Don't wash	63	31
Not reported	3	1.5
**Objects used for intimate washing**		
Finger	112	55.2
Sponge or other	7	3.4
I don’t do this	83	40.9
Not reported	1	0.5
**Products introduced vaginally**		
Yes	17	8.4
No	174	85.7
Not reported	12	5.9

### Type 1 and Type 2 markers of immunity are elevated in the FRT of women positive for hookworm eggs

**Figure 2:**
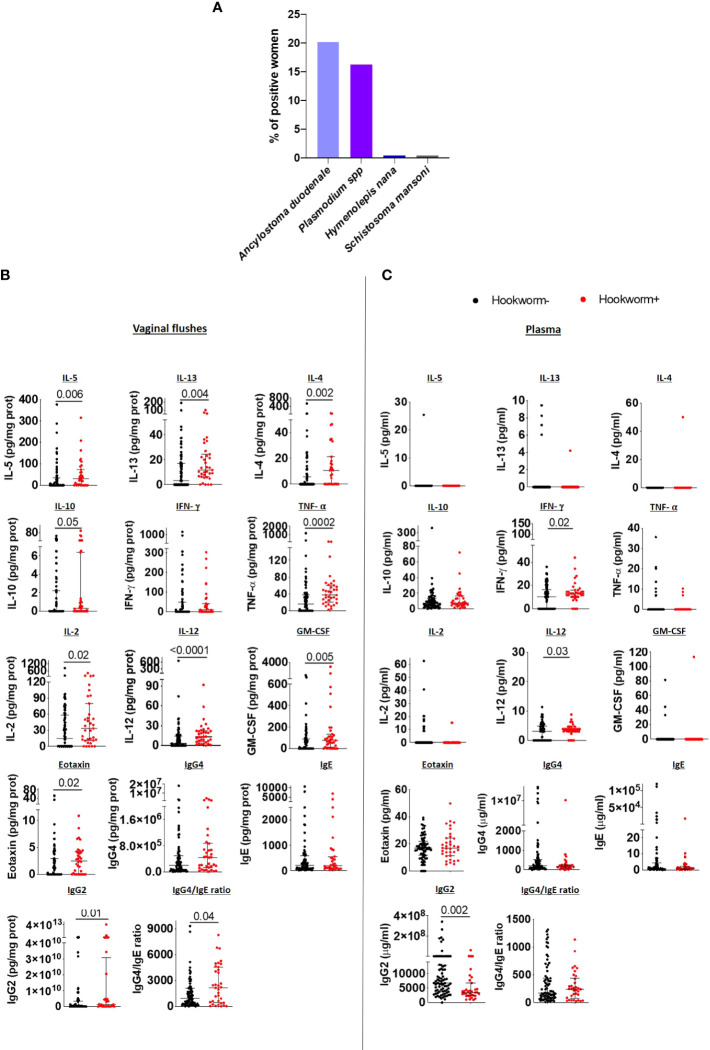
Hookworm infection induces a mixed Type 1/Type 2 immune response in the FRT. **(A)** Overview of parasite infection in the cohort (n=203). Each bar represents the total percentage of women in each category of infection. IL-5, IL-13, IL-4, IL-10, IFN-γ, TNF- α, IL-2, IL-12, GM-CSF, eotaxin, IgG4, IgE and IgG2 levels, and the IgG4/IgE ratio in the vaginal flushes **(B)** and plasma **(C)** of hookworm- and hookworm+ women. Each dot represents a single individual, horizontal bars indicate the median and IQR. To normalize VF data, individual analyte concentrations were expressed as ratio of the measured parameter and the total protein concentration of the sample. Hookworm- (n=78) hookworm+ (n=35). A Mann Whitney U test was used to compare hookworm- and hookworm+ groups.

Among the six villages included in the study, the overall prevalence of hookworm *Ancylostoma duodenale* infection was 20.2% (n=41), followed by *Plasmodium falciparum* (16.3%, n=33) and *Hymenolepis nana* and *Schistosoma mansoni* infections, which were each detected at prevalence of 0.5% (n=1) (**Figure 2A**). Hookworm-infected women demonstrated significantly increased levels of Type 2 immune readouts including raised cytokines IL-5, IL-13, IL-4, IL-10, the chemokine eotaxin, as well as a raised IgG4/IgE ratio in VF when compared to uninfected women. In addition, Type 1 and proinflammatory immune response associated cytokines TNF-α, IL-2, IL-12, GM-CSF as well as IgG2 levels were elevated in VFs of hookworm positive women in comparison to uninfected women (**Figure 2B**). However, inflammasome-associated (IL-18 and IL-1β), IL-6 and Th17 (IL-17A, IL-21, IL22) immune responses were unaltered (**Supplementary Figure 1A**) as were other chemokines, except for MIP-1α (**Supplementary Figure 2B**). Collectively, these findings show that following hookworm infection, Type 1 and Type 2 immune responses are established in the FRT, which supports previous studies demonstrating mixed Th1/Th2 responses in hookworm-infected individuals (17, 18).

However, immune responses in plasma did not correspond to those detected in vaginal samples. In detail, Type 2 and regulatory immune responses did not differ between hookworm+ and hookworm- women and IL-5, IL-13 and IL-4 levels were undetectable in most plasma samples (**Figure 2B**). Only IFN-γ, IL-12 and IL-21 were elevated while IL-18 and IgG2 were reduced in the plasma of hookworm+ women (**Figure 2C**; **Supplementary Figure 1B**). Additionally, chemokines MIP-1α and SDF-1α were elevated whereas RANTES was reduced in hookworm+ women (**Supplementary Figure 2B**). No differences in the vaginal flush and plasma levels of IgA, IgM, IgG1 and IgG3 were observed between hookworm- and hookworm+ women (**Supplementary Figure 3**).

### Hookworm infection positively associated with HPV-DNA load

**Figure 3:**
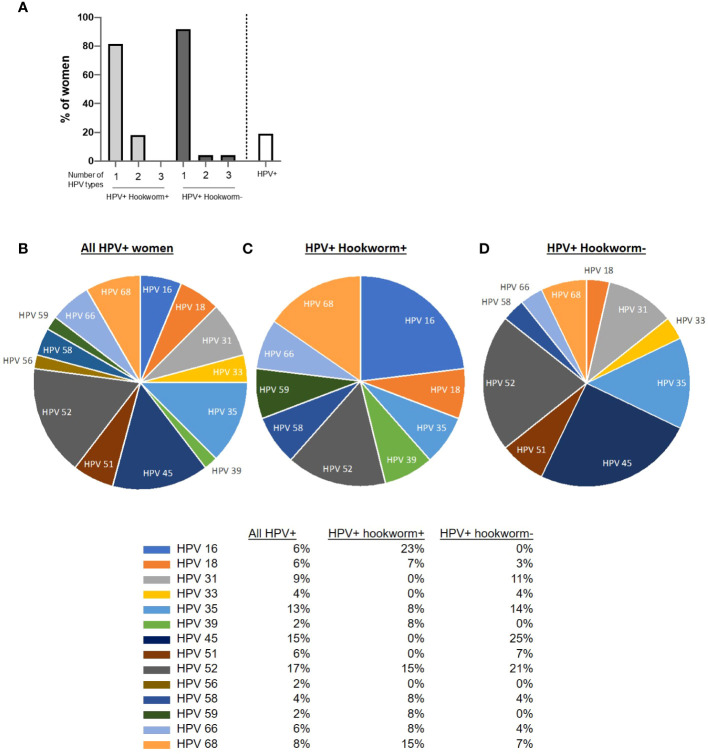
HPV distribution in the cohort. **(A)** Overview of women infected with one or multiple HPV subtypes in the cohort and percentage prevalence of HPV in the cohort. **(B–D)** Pie chart showing the proportion of women positive for the different HPV types tested. Each slice of the pie represents the total percentage of women in each category of infection.

Similar to the percentage hookworm positivity in the cohort, 19.2% of participants sampled were HPV positive (white bar; **Figure 3A**). The majority of women were infected with only one HPV type (82% of HPV+ hookworm+ women and 92% of HPV+ hookworm- women), 18% of HPV+ hookworm+ and 4% of HPV+ hookworm- women were infected with two or three HPV types (**Figure 3A**). The most prevalent HPV types detected were 52, 45 and 35 (17%, 15% and 13%; **Figure 3B**). Globally, approximately 70% of cervical cancer cases are attributable to HPV16 and 18 infection (19). We identified a higher proportion of HPV+ hookworm+ women infected with HPV16 (23%) and 18 (7%) (**Figure 3C** **)** in comparison to HPV+ hookworm– women who had no HPV16 infections and only 3% HPV18 infections (**Figure 3D**). We previously reported that women infected with hookworm were two times more likely to be HPV positive than hookworm-uninfected women (7) and now expand on that finding in this study by showing that hookworm infection is positively associated with an increased intensity of HPV infection (**Table 3**). Interestingly, there was a high prevalence of *Plasmodium* infection in our cohort (16.3%; **Figure 2A**). Therefore, we performed a Chi-square test to determine if *Plasmodium* infection influence the obtained results, but there was no significant association between *Plasmodium* infection and HPV or hookworm infection. *Plasmodium* infection was also not associated with hookworm- HPV-, hookworm- HPV+, hookworm+ HPV-, hookworm+ HPV+ infection status (**Supplementary Table 1**).

**Table 3:** Hookworm infection is positively associated with increased HPV load.

HPV intensity grade	Hookworm infection		
	OR	95%CI	p-value
0	1		
1	2.14	0.52-8.84	0.292
2	1.11	0.23-5.44	0.897
**3**	**3.18**	**1.13-8.98**	0.029*

Bold values indicates significant results.

### HPV infection induces mixed Type 1/Type 2 immune responses in the FRT but systemic Type 1 immune responses

**Figure 4:**
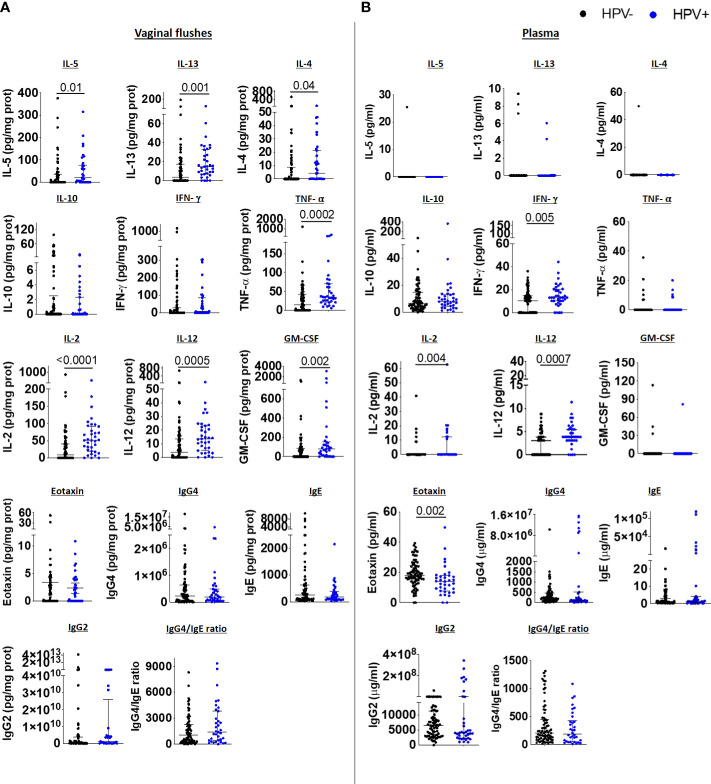
HPV infection induces mixed Type 1/Type 2 immune responses in the FRT accompanied with reduced systemic eotaxin levels. IL-5, IL-13, IL-4, IL-10, IFN-γ, TNF-α, IL-2, IL-12, GM-CSF, eotaxin, IgG4, IgE, IgG2 levels, and IgG4/IgE ratio in vaginal flushes **(A)** and plasma **(B)** of HPV- and HPV+ women. Each dot represents a single individual, horizontal bars indicate the median and IQR. To normalize VF data, individual analyte concentrations were expressed as ratio of the measured parameter and the total protein concentration of the sample. HPV- (n=78) HPV+ (n=35). A Mann Whitney U test was used to compare HPV- and HPV+ groups.

We next compared vaginal immune responses between HPV-infected and uninfected women. Type 2 cytokines IL-5, IL-13, IL-4 were elevated in the VF of HPV-infected women, whereas IL-10, eotaxin and IgE and IgG4 VF levels were comparable to those in HPV- women. Type 1 immune responses TNF-α, IL-2, IL-12 and GM-CSF were also elevated in the VF of HPV-infected compared to HPV negative women (**Figure 4A**). We did not observe any differences in vaginal inflammasome-associated, IL-6 and Th17 immune responses (**Supplementary 4A**), chemokine (**Supplementary Figure 5A**) and antibody levels (**Supplementary Figure 6A**) between HPV+ and HPV- women.

Systemic Type 2 cytokines were undetectable in both HPV+ and HPV- women, while IL-10 levels were comparable between the two groups, and eotaxin levels were decreased in HPV+ women in comparison to HPV- women. However, we detected elevated IFN-γ, IL-2 and IL-12 levels in the plasma of HPV+ (**Figure 4B**), demonstrating that HPV infection induces pronounced systemic Type 1 immune responses and mixed Type 2 and Type 1 immune responses in the FRT. Whereas inflammasome-associated, IL-6 and Th17 immune responses (**Supplementary 4B**) were comparable, the chemokines RANTES, MIP-1β and MCP-1 were significantly decreased in the plasma of HPV-infected women compared to HPV- women (**Supplementary Figure 5B**). Similar to the immunoglobulin data we report in the FRT, systemic antibody levels did not differ between HPV+ and HPV- women (**Supplementary Figure 6B**). In addition, we performed correlation analyses to assess the relationship between HPV intensity and immune parameters measured in the VF and plasma. There were positive correlations between HPV intensity and vaginal flush for IL-2, IL-4, IL-5, IL-12p70, IL-13, GM-CSF and TNF-α levels. In plasma, HPV intensity was negatively correlated with eotaxin, RANTES, MIP-1β and MCP-1 levels and positively with IL-2, IL-12p70, IFN-γ and IgG1 levels (**Supplementary Table 2**).

### Mixed Type 1/Type 2 immune signature is pronounced in the FRT of hookworm and HPV co-infected women

**Figure 5:**
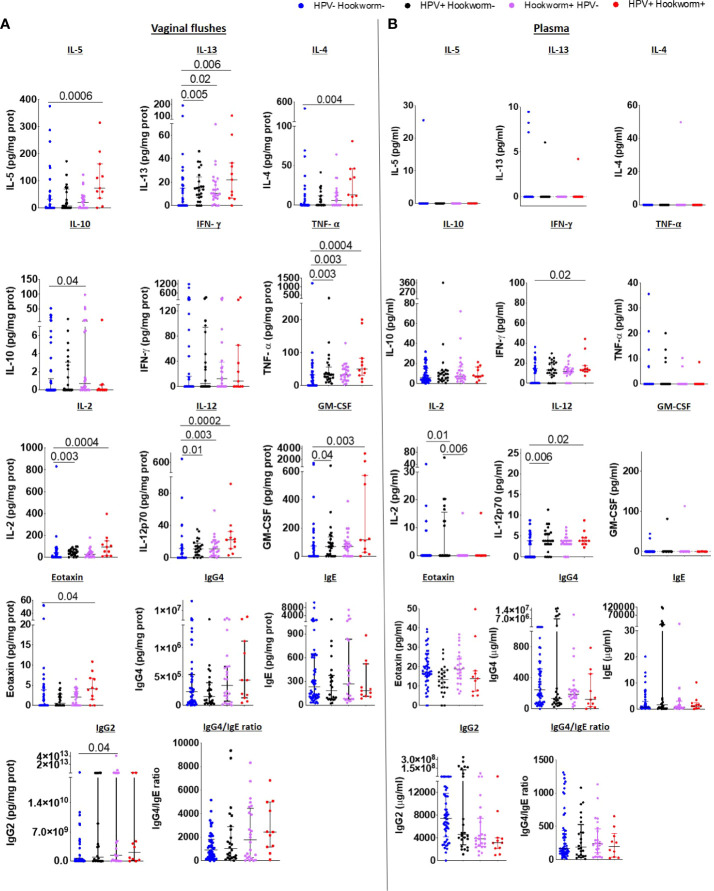
Type 2 immune responses are elevated in the FRT of HPV and hookworm co-infected women IL-5, IL-13, IL-4, IL-10, IFN-γ, TNF-α, IL-2, IL-12, GM-CSF, eotaxin, IgG4, IgE, IgG2 levels, and IgG4/IgE ratio in the vaginal flushes **(A)** and plasma **(B)** of HPV and hookworm negative, single-infected and co-infected women. Each dot represents a single individual, horizontal bars indicate the median and IQR. To normalize VF data, individual analyte concentrations were expressed as ratio of the measured parameter and the total protein concentration of the sample. Hookworm- HPV- (n=54), hookworm- HPV+ (n=24), hookworm+ HPV- (n=24), hookworm+ HPV+ (n=11). Kruskal Wallis with Dunn’s multiple comparisons test were used to compare HPV and hookworm negative, single-infected and co-infected groups.

Since previous studies showed that immunomodulation by helminths can influence susceptibility to viral infection (6, 8–12) and murine hookworm infection exacerbated viral pathology (14), we further compared FRT and systemic immune responses of co-infected women to uninfected and hookworm- and HPV-infected individuals. A pronounced Type 2 immune profile was maintained in the FRT of co-infected women with significantly elevated IL-5, IL-13, IL-4 and eotaxin levels and a trend towards increased IgG4/IgE ratio in co-infected compared to uninfected women. In contrast, women with only hookworm or HPV infection displayed elevated IL-13, but not IL-4, IL-5 or eotaxin levels in the VF. However, a Type1 phenotype was also apparent in the VF of co-infected women, with higher levels of TNF-α, IL-2, IL-12, GM-CSF (**Figure 5A**). Inflammasome-associated, IL-6 and Th17 immune responses (**Supplementary 7A**), chemokine (except MIP-1α;**Supplementary Figure 8A**) and immunoglobulin levels (**Supplementary Figure 9**) in the vaginal flush were comparable between the groups.

Systemic immune responses were not detectable or did not differ significantly among the four groups and only IFN-γ and IL-12 were elevated in the plasma of HPV and hookworm co-infected in comparison to uninfected women (**Figure 5B**). In addition, decreased IL-18 and RANTES were detected in women with only hookworm infection compared to uninfected women, whereas IL-21 and SDF-1α were increased (**Supplementary Figures 7B** and **8B**). Comparable to the vaginal flush, no differences in systemic immunoglobulin levels between co-infected, HPV- or hookworm-infected, or uninfected individuals was observed (**Supplementary Figure 9**).

In summary, this study identifies an induction of mixed Type1/Type 2 immune responses in the FRT in hookworm infected women and this phenotype is also pronounced in HPV and hookworm co-infected women. Finally, increased risk (7) and intensity of HPV infection was identified in hookworm-infected women. These findings provide further proof that helminth infection modulates immune responses distal to the site of infection and thus might be a significant risk factor for sexually transmitted virus infections and present a raised risk for subsequent FRT pathology, such as cancer in the case of HPV.

## Discussion

In this study, we identify increased load of HCR-HPV genome equivalents in hookworm co-infected women from Togo. Due to the integration of viral DNA inside the genome of the epithelial cells, the PCR cannot distinguish the infected epithelial cell and the viral particle. This kind of distinction is possible, but it is not a standardized diagnostic procedure. However, this suggests that a pre-existing helminth infection is a risk factor for cervical cancer and builds on our previous work identifying that hookworm infection is associated with an increased risk of HPV infection in this area (7). We also find hookworm exposure associates with infection with distinct HPV types that are associated with progression to cancer.

Additionally, we identify hookworm infection driving a mixed Type1/Type 2 immune response in the FRT which is an addition to previous studies showing that hookworm infections induce systemic mixed Th1/Type 2 immune responses (17, 18). This signature was more pronounced in women with HPV-hookworm co-infection, which agrees with findings reported by Gravitt et al. (6). However, mixed vaginal Type 1/Type 2 immunity is not widely reported or understood in the context of sexually transmitted infections and its influence may vary depending on the context of infection and study. Of interest is that in hookworm infected women IL-10 is increased in vaginal flushes. However, this increase is not apparent in co-infected women. It could be that in co-infection immune regulation associated with helminth infection is lost promoting a dysregulated Type1/Type 2 inflammatory environment. This would need to be addressed in further studies and would be counter to much literature which supports raised IL-10 as risk factor for cervical cancer (20).

The role of Type 2 immunity in vaginal immunity is currently only addressed in a small number of studies and remains unclear. In *C. trachomatis* infections a Th2 immune response in the uterus has been shown to reduce inflammation and promote tissue repair in the uterus (21, 22). Conversely, co-infection with *N. brasiliensis* and HSV-2 results in a Type 2 FRT signature that drives eosinophil-driven vaginal epithelial necrosis (14). In the current study our findings of raised IL-5 and eotaxin support hookworm infection promoting a vaginal eosinophil response which may promote viral establishment and expansion. These findings differ from existing *in vitro* work addressing helminth influence on HPV infection (23). This suggests a complex relationship underlying helminth and HPV co-infection biology. However, mass drug administration is implemented in Togo and 4 months before enrollment of the study albendazole, ivermectin and praziquantel was distributed to the inhabitants of the Central Region, which might influence the obtained results. Nevertheless, the high prevalence rate of helminths suggests that inhabitants did not receive or took the drugs, or that reinfections and drug resistance are hindering the success of the MDA campaigns.

Of note, we detected Th1/2 responses at a mucosal site (i.e., in vaginal flushes) directly *ex vivo* and independent of immune stimulation, whereas the pronounced Type 1/Type 2 phenotype was not detected in the plasma. Hookworm infections have been shown to induce Type 2 immune responses systemically and in the mucosa of colonised tissue (24) following antigen stimulation. Our findings suggest circulatory immune signatures reflective of distal mucosal infection may not be inherently detectable; instead requiring unmasking by antigen/mitogen stimulation of PBMCs (24). Mucosal immune signatures however are readily detectable independently of antigen/mitogen stimulation. This is likely reflective of sampling a site of ongoing immune challenge. Our data also support a distal helminth infection being able to exert a strong immune footprint on the FRT that is maintained following challenge with an unrelated pathogen, such as HPV.

HPV is a non-lytic virus and infection only occurs when vaginal epithelium is compromised enabling virions to target basal epithelial cells and establish persistent infection (25). Risk factors for HPV infection include other infections which disrupt vaginal epithelium, such as *Chlamydia trachomatis* and HIV (26). Vaginal trauma and injury as a result of sexual activity, and insertion of objects (including cleaning products) into the vagina which cause epithelial microabrasions are also important risk factors for HPV (27). In our study, harsh vaginal cleaning practices were common and in a univariate analysis, the use of water and soap for vaginal cleaning positively associated with increased risk of HPV (7). However, after adjusting for confounders, this association was not significant.

The most prevalent HPV types in our cohort were HPV 52, 45 and 35. Previous studies in Togo, reported HPV types 58, 35 and 16 (28) and HPV types 56, 51, 31, 52 and 35 (29) as most prevalent. In Ghana, Kenya and South Africa however HPV types 16, 52 and 35 (30), HPV types 52, 35 and 51 (31) and HPV types 16, 51, 18 and 35 (32), respectively, were most prevalent. These data indicate that the high-risk HPV types vary by cohort and geographical location. Interestingly, the increased prevalence of HPV 16 and HPV 18 that we observe in hookworm infected in comparison to hookworm uninfected women could imply these women are at a higher risk of cancer, as the majority of cervical cancer cases are attributed to these two HPV types (19).

In summary, this study identifies hookworm infection as a risk factor for an increased load of HPV infection. We also identify hookworm infection as inducing a Type 1/Type 2 immune signature in the vagina and that this is a feature of HPV-hookworm co-infection. This may have important consequences for understanding risk of cervical cancer and moreover, might be taken into consideration for anti-viral treatment as well as helminth deworming programmes. In addition, we detected a distribution of HPV types that has similarities and differences to that reported in some cohorts in Sub-Saharan Africa (SSA). Importantly, HPV 35 that is implicated in cervical cancer progression in SSA (33, 34), and not included in any current HPV vaccine, is one of the predominant HPV types we detected in our cohort. This highlights a need to develop better HPV vaccines specific for each population according to HPV type distribution.

## Data availability statement

The raw data supporting the conclusions of this article will be made available by the authors, without undue reservation.

## Ethics statement

The studies involving human participants were reviewed and approved by Comité de Bioéthique pour la Recherche en Santé (CBRS) of the Ministry of Health of Togo (N°26/2017/CBRS) and the Ethics Committee at the University Hospital Bonn, Bonn, Germany (Lfd. Nr. 273/16). The patients/participants provided their written informed consent to participate in this study.

## Author contributions

GK, LEL, WGCH and MR conceived and designed the study. GK, EHK, CNT, CV, EPT, OMA, KA, SDK, YA and MK performed the field research. MAO, GK, CNT, CV, EPT, OMA, KA, WGCH and MR analyzed the results. MAO, WGCH and MR drafted the manuscript while GK, CNT, KA, MP, LEL and AH critically revised the article and controlled the intellectual content. All authors contributed to the article and approved the submitted version.

## Funding

This work was supported by the German Research Foundation (DFG) within the German-African Projects in Infectiology (GSAT project; LA 2746/2-1). Moreover, MR is financially supported by the German Federal Ministry of Education and Research (BMBF) and the Ministry of Culture and Science of the State of North Rhine-Westphalia (MKW) within the framework of the Excellence Strategy of the Federal and State Governments. AH is supported by the BMBF [01KA1611 and 01KA2027] and additionally funded by the DFG under Germany’s Excellence Strategy – EXC2151 – 390873048. MAO was supported by the Poliomyelitis Research Foundation Bursary (South Africa, Grant No. 18/99). The funders had no role in study design, data collection and analysis, decision to publish, or preparation of the manuscript.

## Acknowledgments

We thank all participants, community health workers as well as local and national health officials for their support. In addition, we thank Mrs. Özlem Mutluer for excellent technical assistance. The Wellcome Centre for Infectious Disease Research in Africa is supported by core funding from the WellcomeTrust (grant 203135/Z/16/Z).

## Conflict of interest

The authors declare that the research was conducted in the absence of any commercial or financial relationships that could be construed as a potential conflict of interest.

## Publisher’s note

All claims expressed in this article are solely those of the authors and do not necessarily represent those of their affiliated organizations, or those of the publisher, the editors and the reviewers. Any product that may be evaluated in this article, or claim that may be made by its manufacturer, is not guaranteed or endorsed by the publisher.

## References

[1] WHO. (2020). World Health Organization. Available at: https://apps.who.int/neglected_diseases/ntddata/sth/sth.html.

[2] Chetty A Omondi MA Butters C Smith KA Katawa G Ritter M. Impact of helminth infections on female reproductive health and associated diseases. Front Immunol (2020) 11:1–13. doi: 10.3389/fimmu.2020.577516 33329545 PMC7719634

[3] Osborne LC Monticelli LA Nice TJ Sutherland TE Siracusa MC Hepworth MR. Virus-helminth coinfection reveals a microbiota-independent mechanism of immunomodulation. Sci (80- ) (2014) 345(6196):578–82. doi: 10.1126/science.1256942 PMC4548887 25082704

[4] Reese TA Wakeman BS Choi HS Hufford MM Huang SC Zhang X. Helminth infection reactivates latent γ-herpesvirus *via* cytokine competition at a viral promoter. Sci (80- ) (2014) 345(6196):573–7. doi: 10.1126/science.1254517 PMC4531374 24968940

[5] Rolot M Dougall AM Chetty A Javaux J Chen T Xiao X. Helminth-induced IL-4 expands bystander memory CD8+ T cells for early control of viral infection. Nat Commun (2018) 9(1):4516. doi: 10.1038/s41467-018-06978-5 30375396 PMC6207712

[6] Gravitt PE Marks M Kosek M Huang C Cabrera L Olortegui MP. Soil-transmitted helminth infections are associated with an increase in human papillomavirus prevalence and a T-helper type 2 cytokine signature in cervical fluids. J Infect Dis (2016) 212(11):723–30. doi: 10.1093/infdis/jiv498 PMC4747620 26486638

[7] Holali Ameyapoh A Katawa G Ritter M Tchopba CN Tchadié PE Arndts K. Hookworm infections and sociodemographic factors associated with female reproductive tract infections in rural areas of the central region of Togo. Front Microbiol (2021) 12:738894/full. doi: 10.3389/fmicb.2021.738894/full 34803955 PMC8595254

[8] Odegaard JI Hsieh MH. Immune responses to schistosoma haematobium infection. Parasite Immunol (2014) 36(9):428–38. doi: 10.1111/pim.12084 25201406

[9] Kjetland EF Ndhlovu PD Gomo E Mduluza T Midzi N Gwanzura L. Association between genital schistosomiasis and HIV in rural Zimbabwean women. AIDS (2006) 20(4):593–600. doi: 10.1097/01.aids.0000210614.45212.0a 16470124

[10] Ndhlovu PD Mduluza T Kjetland EF Midzi N Nyanga L Gundersen SG. Prevalence of urinary schistosomiasis and HIV in females living in a rural community of Zimbabwe: does age matter? Trans R Soc Trop Med Hyg (2007) 101(5):433–8. doi: 10.1016/j.trstmh.2006.08.008 17064746

[11] Downs JA Mguta C Kaatano GM Mitchell KB Bang H Simplice H. Urogenital schistosomiasis in women of reproductive age in tanzania’s lake Victoria region. Am J Trop Med Hyg (2011) 84(3):364–9. doi: 10.4269/ajtmh.2011.10-0585 PMC3042809 21363971

[12] Kroidl I Chachage M Mnkai J Nsojo A Berninghoff M Verweij JJ. Wuchereria bancrofti infection is linked to systemic activation of CD4 and CD8 T cells. babu s, editor. PloS Negl Trop Dis (2019) 13(8):e0007623. doi: 10.1371/journal.pntd.0007623 31425508 PMC6736309

[13] Smith JS Herrero R Bosetti C Muñoz N Bosch FX Eluf-Neto J. Herpes simplex virus-2 as a human papillomavirus cofactor in the etiology of invasive cervical cancer. J Natl Cancer Inst (2002) 94(21):1604–13. doi: 10.1093/jnci/94.21.1604 12419786

[14] Chetty A Darby MG Vornewald PM Martín-Alonso M Filz A Ritter M. Il4ra-independent vaginal eosinophil accumulation following helminth infection exacerbates epithelial ulcerative pathology of HSV-2 infection. Cell Host Microbe (2021) 29(4):579–593.e5. doi: 10.1016/j.chom.2021.02.004 33857419 PMC8062792

[15] Katz N A. Pellegrino J. A simple device for quantitative stool thick smear technique in schistosomiasis mansoni. Rev Soc Bras Med Trop (1972) 14:397–400. 4675644

[16] Oštrbenk A Xu L Arbyn M Poljak M. Clinical and analytical evaluation of the anyplex II HPV HR detection assay within the VALGENT-3 framework. loeffelholz MJ, editor. J Clin Microbiol (2018) 56(11):1–8. doi: 10.1128/JCM.01176-18 PMC6204677 30209184

[17] Geiger SM Caldas IR Mc Glone BE Campi-Azevedo AC De Oliveira LM Brooker S. Stage-specific immune responses in human necator americanus infection. Parasite Immunol (2007) 29(7):347–58. doi: 10.1111/j.1365-3024.2007.00950.x PMC1976388 17576364

[18] Quinnell RJ Bethony J Pritchard DI. The immunoepidemiology of human hookworm infection. Parasite Immunol (2004) 26(11–12):443–54. doi: 10.1111/j.0141-9838.2004.00727.x 15771680

[19] de Sanjose S Quint WGV Alemany L Geraets DT Klaustermeier JE Lloveras B. Human papillomavirus genotype attribution in invasive cervical cancer: a retrospective cross-sectional worldwide study. Lancet Oncol (2010) 11(11):1048–56. doi: 10.1016/S1470-2045(10)70230-8 20952254

[20] Berti FCB Pereira APL Cebinelli GCM Trugilo KP Brajão de Oliveira K. The role of interleukin 10 in human papilloma virus infection and progression to cervical carcinoma. Cytokine Growth Factor Rev [Internet] (2017) 34:1–13. doi: 10.1016/j.cytogfr.2017.03.002 28365229

[21] Vicetti Miguel RD Harvey SAK LaFramboise WA Reighard SD Matthews DB Cherpes TL. Human female genital tract infection by the obligate intracellular bacterium chlamydia trachomatis elicits robust type 2 immunity. coers J, editor. PloS One (2013) 8(3):e58565. doi: 10.1371/journal.pone.0058565 23555586 PMC3603585

[22] Vicetti Miguel RD Quispe Calla NE Dixon D Foster RA Gambotto A Pavelko SD. IL-4–secreting eosinophils promote endometrial stromal cell proliferation and prevent chlamydia-induced upper genital tract damage. Proc Natl Acad Sci U S A (2017) 114(33):E6892–901. doi: 10.1073/pnas.1621253114 PMC5565408 28765368

[23] Jacobs BA Chetty A Horsnell WGC Schäfer G Prince S Smith KA. Hookworm exposure decreases human papillomavirus uptake and cervical cancer cell migration through systemic regulation of epithelial-mesenchymal transition marker expression. Sci Rep (2018) 8(1):2–10. doi: 10.1038/s41598-018-30058-9 30069018 PMC6070561

[24] Gaze S McSorley HJ Daveson J Jones D Bethony JM Oliveira LM. Characterising the mucosal and systemic immune responses to experimental human hookworm infection. PloS Pathog (2012) 8(2):e1002520. doi: 10.1371/journal.ppat.1002520 22346753 PMC3276555

[25] Stubenrauch F Laimins LA. Human papillomavirus life cycle: active and latent phases. Semin Cancer Biol (1999) 9(6):379–86. doi: 10.1006/scbi.1999.0141 10712884

[26] Samoff E Koumans EH Markowitz LE Sternberg M Sawyer MK Swan D. Association of chlamydia trachomatis with persistence of high-risk types of human papillomavirus in a cohort of female adolescents. Am J Epidemiol (2005) 162(7):668–75. doi: 10.1093/aje/kwi262 16120706

[27] Cottrell BH. An updated review of of evidence to discourage douching. MCN Am J Matern Nurs (2010) 35(2):102–7. doi: 10.1097/NMC.0b013e3181cae9da 20215951

[28] Ferré VM Ekouevi DK Gbeasor-Komlanvi FA Collin G Le Hingrat Q Tchounga B. Prevalence of human papillomavirus, human immunodeficiency virus and other sexually transmitted infections among female sex workers in Togo: a national cross-sectional survey. Clin Microbiol Infect (2019) 25(12):1560.e1–1560.e7. doi: 10.1016/j.cmi.2019.04.015 31051265

[29] Kuassi-Kpede AP Dolou E Zohoncon TM Traore IMA Katawa G Ouedraogo RA. Molecular characterization of high-risk human papillomavirus (HR-HPV) in women in lomé, Togo. BMC Infect Dis (2021) 21(1):1–7. doi: 10.1186/s12879-021-05956-5 33740909 PMC7977574

[30] Krings A Dunyo P Pesic A Tetteh S Hansen B Gedzah I. Characterization of human papillomavirus prevalence and risk factors to guide cervical cancer screening in the north tongu district, Ghana. PloS One (2019) 14(6):1–19. doi: 10.1371/journal.pone.0218762 PMC6597158 31246997

[31] Sweet K Bosire C Sanusi B Sherrod CJ Kwatampora J Waweru W. Prevalence, incidence, and distribution of human papillomavirus types in female sex workers in Kenya. Int J STD AIDS (2020) 31(2):109–18. doi: 10.1177/0956462419884454 PMC7031817 31948341

[32] Mbatha JN Taylor M Kleppa E Lillebo K Galapaththi-Arachchige HN Singh D. High-risk human papillomavirus types in HIV-infected and HIV-uninfected young women in KwaZulu-natal, south Africa: implications for vaccination. Infect Dis (Auckl) (2017) 49(8):601–8. doi: 10.1080/23744235.2017.1312513 28403727

[33] Carlander C Lagheden C Eklund C Kleppe SN Dzabic M Wagner P. HPV types in cervical precancer by HIV status and birth region: A population-based register study. Cancer Epidemiol Biomarkers Prev (2020) 29(12):2662–8. doi: 10.1158/1055-9965.EPI-20-0969 32967862

[34] Pinheiro M Gage JC Clifford GM Demarco M Cheung LC Chen Z. Association of HPV35 with cervical carcinogenesis among women of African ancestry: Evidence of viral-host interaction with implications for disease intervention. Int J Cancer (2020) 147(10):2677–86. doi: 10.1002/ijc.33033 PMC11090644 32363580

