# Supplementary material for: Hookworm infection associates with a vaginal Type 1/Type 2 immune signature and increased HPV load
Source: Front Immunol. 2022 Oct 18;13:1009968. doi: 10.3389/fimmu.2022.1009968 (PMC9623172; doi:10.3389/fimmu.2022.1009968)
Supplement: Supplementary file 1 [file DataSheet_1.docx]

Supplementary Material

**Supplementary Table 1:** Influence of *Plasmodium* infection on hookworm- HPV-, hookworm- HPV+, hookworm+ HPV-, hookworm+ HPV+ infection status. P-values denote statistical differences between the groups for that parameter tested by Chi-square test

| ***Plasmodium spp.*** | **No** | **Yes** | **p-value** |
| --- | --- | --- | --- |
|  |  |  |  |
| **HPV** |  |  | 0.089 |
| No | 61 (65.6%) | 17 (85%) |  |
| Yes | 32 (34.4%) | 3 (15%) |  |
|  |  |  |  |
| **Hookworm** |  |  | 0.668 |
| No | 65 (69.9%) | 13 (65%) |  |
| Yes | 28 (30.1%) | 7 (35%) |  |
|  |  |  |  |
| **Single or co-infected** |  |  | 0.061 |
| Hookworm^-^ HPV^-^ | 41 (44.1%) | 13 (65%) |  |
| Hookworm^-^ HPV^+^ | 24 (25.8%) | 0 (0%) |  |
| Hookworm^+^ HPV^-^ | 20 (21.5%) | 4 (20%) |  |
| Hookworm^+^ HPV^+^ | 8 (8.6%) | 3 (15%) |  |

**Supplementary Table 2:** Correlation test of the relationship between HPV intensity and immune parameters measured in the vaginal flushes and plasma. P-values <0.05 were considered statistically significant and are indicated in bold

|  | **Vaginal Flushes** | | **Plasma** | |
| --- | --- | --- | --- | --- |
|  | Regression co-efficient | p value | Regression co-efficient | p value |
| MIP-1⍺ | 0.161 | 0.089 | -0.133 | 0.163 |
| SDF-1⍺ | -0.097 | 0.309 | -0.053 | 0.58 |
| IL-1β | 0.048 | 0.610 | 0.077 | 0.421 |
| IL-2 | 0.413 | **<0.001** | 0.275 | **0.003** |
| IL-4 | 0.195 | **0.038** | -0.063 | 0.511 |
| IL-5 | 0.27 | **0.004** | -0.063 | 0.511 |
| IP-10 | -0.05 | 0.597 | -0.154 | 0.105 |
| IL-6 | 0.062 | 0.517 | -0.049 | 0.607 |
| IL-8 | -0.092 | 0.331 | -0.148 | 0.120 |
| Eotaxin | 0.095 | 0.317 | -0.275 | **0.003** |
| IL-12 | 0.333 | **<0.001** | 0.312 | **0.001** |
| IL-13 | 0.308 | **0.001** | 0 | 0.999 |
| RANTES | 0.149 | 0.116 | -0.263 | **0.006** |
| IFN-γ | 0.13 | 0.170 | 0.245 | **0.009** |
| GM-CSF | 0.289 | **0.002** | -0.03 | 0.753 |
| TNF-⍺ | 0.335 | **<0.001** | 0.06 | 0.531 |
| MIP-1β | -0.124 | 0.192 | -0.260 | **0.006** |
| MCP-1 | -0.121 | 0.201 | -0.192 | **0.043** |
| GRO-⍺ | -0.029 | 0.761 | -0.030 | 0.753 |
| IL-18 | 0.022 | 0.818 | -0.092 | 0.337 |
| IL-10 | -0.048 | 0.612 | 0.063 | 0.51 |
| Eotaxin-2 | -0.064 | 0.501 | 0.119 | 0.214 |
| Eotaxin-3 | 0.078 | 0.417 | -0.044 | 0.645 |
| IL-17 | 0.054 | 0.571 | -0.053 | 0.584 |
| IL-21 | 0.042 | 0.665 | 0.114 | 0.234 |
| IL-22 | 0.098 | 0.307 | 0.058 | 0.545 |
| IgG1 | -0.138 | 0.145 | 0.491 | **0.039** |
| IgG2 | 0.082 | 0.388 | -0.109 | 0.251 |
| IgG3 | -0.107 | 0.261 | 0.126 | 0.425 |
| IgG4 | 0.003 | 0.974 | -0.118 | 0.217 |
| IgE | -0.096 | 0.312 | 0.086 | 0.366 |
| IgA | 0.013 | 0.889 | 0.047 | 0.625 |
| IgM | -0.09 | 0.344 | 0.043 | 0.665 |
|  |  |  |  |  |

**Supplementary Figure 1: Inflammasome-associated IL-6 and Th17 immune responses in vaginal flush and plasma of hookworm- and hookworm+ women.** IL-18, IL-6, IL-1β, IL-17A, IL-21 and IL-22 levels in the vaginal flushes (A) and plasma (B) of hookworm- and hookworm+ women. Each dot represents a single individual, horizontal bars indicate the median and IQR. To normalize VF data, individual analyte concentrations were expressed as ratio of the measured parameter and the total protein concentration of the sample. Hookworm- (n=78) hookworm+ (n=35). A Mann Whitney U test was used to compare hookworm- and hookworm+ groups.

**Supplementary Figure 2: Chemokine levels in vaginal flush and plasma of hookworm- and hookworm+ women.**

RANTES, IL-8, MIP-1⍺, MIP-1β, MCP-1, GRO-⍺, SDF-1⍺, IP-10, eotaxin-2 and eotaxin-3 levels in vaginal flushes (A) and plasma (B) of hookworm- and hookworm+ women. Each dot represents a single individual, horizontal bars indicate the median and IQR. To normalize VF data, individual analyte concentrations were expressed as ratio of the measured parameter and the total protein concentration of the sample. Hookworm- (n=78) hookworm+ (n=35). A Mann Whitney U test was used to compare hookworm- and hookworm+ groups.

**Supplementary Figure 3: Immunoglobulin levels in vaginal flush and plasma of hookworm- and hookworm+ women.** IgA, IgM, IgG1 and IgG3 levels in vaginal flushes (A) and plasma (B) of hookworm- and hookworm+ women. Each dot represents a single individual, horizontal bars indicate the median and IQR. To normalize VF data, individual analyte concentrations were expressed as ratio of the measured parameter and the total protein concentration of the sample. Hookworm- (n=78) hookworm+ (n=35). A Mann Whitney U test was used to compare hookworm- and hookworm+ groups.

**Supplementary Figure 4: Inflammasome-associated, IL-6 and Th17 immune responses in vaginal flush and plasma of HPV- and HPV+ women.** IL-18, IL-6, IL-1β, IL-17A, IL-21 and IL-22 levels in the vaginal flushes (A) and plasma (B) of HPV- and HPV+ women. Each dot represents a single individual, horizontal bars indicate the median and IQR. To normalize VF data, individual analyte concentrations were expressed as ratio of the measured parameter and the total protein concentration of the sample. Hookworm- (n=78) hookworm+ (n=35). A Mann Whitney U test was used to compare HPV- and HPV+ groups.

**Supplementary Figure 5: Chemokine levels in vaginal flush and plasma of hookworm- and hookworm+ women.**

Eotaxin, RANTES, IL-8, MIP-1⍺, MIP-1β, MCP-1, GRO-⍺, SDF-1⍺, IP-10, eotaxin-2 and eotaxin-3 levels in vaginal flushes (A) and plasma (B) of HPV- and HPV+ women. Each dot represents a single individual, horizontal bars indicate the median and IQR. To normalize VF data, individual analyte concentrations were expressed as ratio of the measured parameter and the total protein concentration of the sample. HPV- (n=78) HPV+ (n=35). A Mann Whitney U test was used to compare HPV- and HPV+ groups.

**Supplementary Figure 6: Immunoglobulin levels in vaginal flush and plasma of hookworm- and hookworm+ women.**

IgA, IgM, IgG1 and IgG3 levels in vaginal flushes (A) and plasma (B) of HPV- and HPV+ women. Each dot represents a single individual, horizontal bars indicate the median and IQR. To normalize VF data, individual analyte concentrations were expressed as ratio of the measured parameter and the total protein concentration of the sample. HPV- (n=78) HPV+ (n=35). A Mann Whitney U test was used to compare HPV- and HPV+ groups.

**Supplementary Figure 7: Inflammasome-associated, IL-6 and Th17 immune responses in vaginal flush and plasma of hookworm-HPV co-infected women.** IL-18, IL-6, IL-1β, IL-17A, IL-21 and IL-22 levels in the vaginal flushes (A) and plasma (B) of HPV and hookworm negative, single-infected and co-infected women. Each dot represents a single individual, horizontal bars indicate the median and IQR. To normalize VF data, individual analyte concentrations were expressed as ratio of the measured parameter and the total protein concentration of the sample. Hookworm- HPV- (n=54), hookworm- HPV+ (n=24), hookworm+ HPV- (n=24), hookworm+ HPV+ (n=11). Kruskal Wallis with Dunn’s multiple comparisons test was used to compare HPV and hookworm negative, single-infected and co-infected groups.

**Supplementary Figure 8: Chemokine levels in vaginal flush and plasma of hookworm and HPV co-infected women.** RANTES, IL-8, MIP-1⍺, MIP-1β, MCP-1, GRO-⍺, SDF-1⍺, IP-10, eotaxin-2 and eotaxin-3 levels in vaginal flushes (A) and plasma (B) levels in vaginal flushes of HPV and hookworm negative, single-infected and co-infected women. Each dot represents a single individual, horizontal bars indicate the median and IQR. To normalize VF data, individual analyte concentrations were expressed as ratio of the measured parameter and the total protein concentration of the sample. Hookworm- HPV- (n=54), hookworm- HPV+ (n=24), hookworm+ HPV- (n=24), hookworm+ HPV+ (n=11). Kruskal Wallis with Dunn’s multiple comparisons test was used to compare HPV and hookworm negative, single-infected and co-infected groups.

**Supplementary Figure 9: Immunoglobulin levels in vaginal flush and plasma of hookworm and HPV co-infected women.** IgA, IgM, IgG1 and IgG3 levels in vaginal flushes (A) and plasma (B) of HPV and hookworm negative, single-infected and co-infected women. Each dot represents a single individual, horizontal bars indicate the median and IQR. To normalize VF data, individual analyte concentrations were expressed as ratio of the measured parameter and the total protein concentration of the sample. Hookworm- HPV- (n=54), hookworm- HPV+ (n=24), hookworm+ HPV- (n=24), hookworm+ HPV+ (n=11). Kruskal Wallis with Dunn’s multiple comparisons test was used to compare HPV and hookworm negative, single-infected and co-infected groups.
